# Local expression profiles of vitamin D-related genes in airways of COPD patients

**DOI:** 10.1186/s12931-020-01405-0

**Published:** 2020-06-03

**Authors:** Carolien Mathyssen, Celine Aelbrecht, Jef Serré, Stephanie Everaerts, Karen Maes, Ghislaine Gayan-Ramirez, Bart Vanaudenaerde, Wim Janssens

**Affiliations:** 1 Department CHROMETA, Laboratory of Respiratory diseases and Thoracic Surgery (BREATHE), KU Leuven, Leuven, Belgium; 2 Clinical department of Respiratory Diseases, UZ Leuven, Campus Gasthuisberg, ON I Herestraat 49 - bus, 706 3000 Leuven, Belgium

**Keywords:** COPD, Vitamin D, Vitamin D receptor, CYP27B1, CYP24A1, Cathelicidin

## Abstract

Treatment of Chronic Obstructive Pulmonary Disease (COPD) is based on bronchodilation, with inhaled corticosteroids or azithromycin associated when frequent exacerbations occur. Despite the proven benefits of current treatment regimens, the need for new interventions in delineated subgroups remains. There is convincing evidence for oral vitamin D supplementation in reducing exacerbations in COPD patients severely deficient for circulating vitamin D. However, little is known about local vitamin D metabolism in the airways and studies examining expression of the vitamin D receptor (VDR), the activating enzyme (CYP27B1) and inactivating enzyme (CYP24A1) of vitamin D in lung tissue of COPD patients are lacking. Therefore, the expression and localization of key enzymes and the receptor of the vitamin D pathway were examined in tissue of 10 unused donor lungs and 10 COPD explant lungs. No differences in the expression of CYP27B1 and CYP24A1 were found. Although protein expression of VDR was significantly lower in COPD explant tissue, there was no difference in downstream expression of the antimicrobial peptide cathelicidin. Whereas CYP27B1 and CYP24A1 were present in all layers of the bronchial epithelium, VDR was only expressed at the apical layer of a fully differentiated bronchial epithelium with no expression in vascular endothelial cells. By contrast, CYP24A1 expression was highly present in lung endothelial cells suggesting that systemic vitamin D can be inactivated before reaching the epithelial compartment and the tissue immune cells. These data support the idea of exploring the role of vitamin D inhalation in patients with COPD.

## Background

Chronic Obstructive Pulmonary Disease (COPD) is a chronic lung disease characterized by a progressive airflow limitation due to chronic inflammation of the small airways and destruction of the alveoli. The disease is further complicated by exacerbations which often necessitate a change in medication [1]. Patients with severe exacerbations are at risk for hospitalization and the mortality associated with admission may rise to more than 20% in the first year after discharge [2]. Current therapies mainly focus on bronchodilation by using combined therapies of long-acting β_2_-adrenergic agonists and anticholinergics [1]. Inhaled corticosteroids or azithromycin are used to decrease exacerbation frequency however, effectiveness is more pronounced in specific subgroups [3, 4]. As exacerbations are driving symptoms and compose a significant burden on our healthcare system, there is an obvious need for more powerful anti-inflammatory and immuno-modulating agents [5].

Vitamin D regulates about 3% of the genome and has an important role in calcium and phosphorus homeostasis but also in immunity [6, 7]. Vitamin D is known to interact with the NFkB and p38/MAPK pathway, to inhibit transcription of cytokines and chemokines and to reduce inflammation [8]. Moreover, a vitamin D responsive element is also key in the transcriptional upregulation of the antimicrobial peptide cathelicidin, one of the main antibacterial peptides [9, 10]. Because of its immune modulating effects, multiple studies have tested the effect of oral vitamin D supplementation in COPD. A recent meta-analysis using individual patient data revealed that oral vitamin D supplementation reduced exacerbation frequency by 45% in COPD patients with baseline 25(OH)D levels < 10 ng/ml [11].

Multiple studies have focused on the clinical benefits of vitamin D supplementation in COPD, but despite the observation that dosing and baseline 25(OH)D levels are critical determinants of response, there is little evidence on the local vitamin D levels that one needs to target, nor on its local effects of airway inflammation. As far as we know, studies that carefully addressed expression profiles of vitamin D metabolic genes and key proteins like the vitamin D receptor (VDR), CYP27B1 (1α-hydroxylase, vitamin D activating enzyme) and CYP24A1 (24-hydroxylase, vitamin D inactivation) in COPD (and controls) are lacking. Moreover, localization of VDR, CYP27B1 and CYP24A1 within the lung is not completely understood, even though in vitro studies have demonstrated its functional presence in human airway epithelial cells and alveolar macrophages [12, 13].

Obviously, expression and localization of VDR, CYP27B1 and CYP24A1 within the COPD lungs should be considered important as alterations may explain the individual effect of vitamin D supplementation, as well as the required dose and preferential route of administration. Therefore, we analyzed the expression of VDR, CYP27B1 and CYP24A1 in tissue from control and COPD explant lungs via qRT-PCR, western blot and immunohistochemistry. We also included expression of cathelicidin as a direct readout for functional vitamin D signaling through VDR [14–16].

## Materials and methods

### Tissue collection and sampling

Lungs were collected, the mainstem bronchus was connected to air supply and the lung was inflated at 30 mmH_2_O to total lung capacity. Pressure was reduced to 10 mmH_2_O and lungs were frozen near total lung capacity in the vapors of liquid nitrogen and stored at − 80 °C. Afterwards, lungs were cut in slices of 2 cm thick from apex to base after which cores of 1.4 cm in diameter were pinched. For this study, 10 COPD explant lungs and unused donor lungs were used. One core per lung was used and after microCT and segmentation analysis, it was divided into 3 parts for qRT-PCR, western blotting and immunohistochemistry/immunofluorescence as detailed thereafter. This study was approved by the University Hospital Ethics Committee (S7742, S52174) and biobank board (S51577). Informed consent was obtained from all patients.

### Patient characteristics

Ten COPD explant lungs and unused donor lungs matched for age and gender were included. Unused donor lungs were from never smokers, while all explants from COPD patients had an important smoking history. Table 1 summarizes the clinical baseline characteristics of the groups. Median FEV_1_% of 28.5%, DLCO% of 41% and reduced body weight in the COPD group are illustrative for the lung transplant setting. COPD patients were slightly insufficient for vitamin D (serum 25(OH)D < 30 ng/ml), 25(OH)D levels were missing in 3 COPD patients and eight out of 10 COPD patients received oral vitamin D supplementation. Unfortunately, 25(OH)D levels were not available in the control group.

**Table 1:** Patient characteristics (*n* = 10/group)

	Donor	COPD	***p***-value
**Age**	57 (55–69)	60.5 (54–62)	0.41
**Gender (Male/Female)**	7/3	7/3	> 0.99
**Smoking history (Yes/No)**	0/10^b^	10/0	**< 0.0001**
**Height (cm)**	170 (167–180)	165 (159–174)	0.084
**Weight (kg)**	75 (65–90)	62 (53–74)	**0.019**
**BMI (kg/m**^**2**^**)**	25 (24–28)	23 (20–26)	0.057
**FEV1%**	NA	28,5 (23–36)	NA
**FVC%**	NA	73 (64–87)	NA
**FEV1%/FVC%**	NA	32 (27–37)	NA
**DLCO%**	NA	41 (35–54)^a^	NA
**TLC%**	NA	123 (118–140)	NA
**serum 25(OH)D (μg/L)**	NA	26 (12–30)^a^	NA
**Vit D supplementation (Yes/No/NA)**	0/0/10	8/2/0	NA

^a^Pretransplant 25(OH)D levels and DLCO% were unknown for 3 COPD patients. ^b^ Donors were registered as nonsmokers at the time of allocation Abbreviations: BMI: body mass index, FEV_1_% % predicted forced expiratory volume in 1 s, FVC%: % predicted forced vital capacity, FEV1%/FVC: Tiffeneau index, DLco%: % predicted diffusion capacity, TLC%, % predicted total lung capacity. Values are Median with IQR. Depending on the normality of the data, either an unpaired t-test or a Mann-Whitney U test was used

### μCT and segmentation

MicroCT (Brüker Skyscan 1172 micro CT device, Brüker, Kontich, Belgium) was used to obtain high-resolution images of the cores, which could then be used to determine disease severity and segmentation of the airways. Scans were taken at 40 kV, 240 mA and a rotation step of 0.5° with a resolution of 10 μm at − 30 °C using a Thermostage (Brüker) and a Styrofoam holder. Reconstruction of the μCT scans was done using NRecon (Brüker) and surface area/volume (surface density) was analyzed using CTAn (Brüker) using a manual threshold. Airway segmentation was done in ITK SNAP [17].

### qRT-PCR

Samples were homogenized in TRIzol (Invitrogen, Merelbeke, Belgium) using the Ultra-Turrax (IKA, Staufen, Germany). Afterwards, the homogenate was transferred into eppendorfs and centrifuged at 4 °C for 15 min at 12000 g. The supernatant was then transferred into fresh eppendorfs and stored at − 80 °C until RNA extraction. RNA was extracted using the TRIzol-Chloroform method and by using the RNAeasy kit (Qiagen, Venlo, The Netherlands) using the manufacturers instruction and by using DNase I to remove DNA from the sample. One μg RNA was used for reverse transcription into cDNA using the Superscript III (Invitrogen) kit. qRT-PCR was performed using the Platinum Sybr Green mix (Invitrogen) in a total volume of 10 μl. Results were analyzed using the comparative cycle threshold method and normalized to the geomean of the housekeeping genes GAPDH and β-actin. Primers can be found in Table 2.

**Table 2:** Primer list

Gene	Forward Primer	Reverse primer
GAPDH	TGGTATCGTGGAAGGACTCA	CCAGTAGAGGCAGGGATGAT
β-actin	GCACATCCGAAAGACCTGT	CTCAGGAGGAGCAATGAT
VDR	GATTGGAGAAGCTGGACGAG	GTTCGTGTGAATGATGGTGGA
CYP27B1	CGCACTGTCCCAAAGCTG	CGGAGCTTGGCAGACATC
CYP24A1	GTGACCATCATCCTCCCAAA	AGTATCTGCCTCGTGTTGTATG
Cathelicidin	GGGCTCCTTTGACATCAGTT	AGCAGGGCAAATCTCTTGTT

*Abbreviations*: *GAPDH* Glyceraldehyde-3-phosphate dehydrogenase, *VDR* Vitamin D receptor, *CYP27B1* 1alpha- hydroxylase, *CYP24A1* 24-hydroxylase

### Western blot

Core samples were homogenized using the Ultra-Turrax (IKA) in 1 ml RIPA buffer supplemented with 1 complete mini antiprotease tablet/10 ml (Roche, Basel, Switserland) and 1 PhosSTOP tablet/10 ml (Roche). Samples were transferred in an eppendorf and centrifuged for 15 min at 12000 g at 4 °C. Supernatant was transferred into a new eppendorf, aliquoted and stored at − 80 °C. Protein concentration was measured using the BCA protein assay (Thermofisher Scientific, Merelbeke, Belgium). 50 μg protein was loaded onto a 8% BOLT BIS-TRIS gel (Invitrogen) and the gel was run in MOPS buffer for 45 min at 165 V. Proteins were transferred onto a membrane using the iBLOT 2 system (Invitrogen) and iBLOT 2 regular transfer stacks. as follows: 20 V for 1 min 23 V for 4 min 25 V for 2 min. Afterwards, the membrane was washed 1x with 0.1% Tween/Tris-buffered saline (TBST), blocked for 1 h at room temperature in 5% non fat dry milk (NFDM)/TBST and incubated overnight with the primary antibody diluted 1/1000 or 1/100 for cathelicidin (GAPDH: cell Signalling, VDR (D-6), CYP24A1 (E-7) and cathelicidin (D-5): Santa-Cruz biotechnology, Heidelberg, Germany CYP27B1 (EPR20271): Abcam, Cambridge, UK) in 5%NFDM/TBST. Membranes were then washed 3 times for 5 min with TBST after which the membrane was incubated with the secondary antibody (Rabbit anti-mouse (DAKOP0260) and swine anti-rabbit (DAKOP0217) depending in the primary antibody, Agilent) for 1 h at room temperature. Membranes were washed again 3 times for 5 min after which the proteins were visualized using the ECL prime western blotting system (GE Healthcare) on the Proxima (Isogen life sciences, Utrecht, The Netherlands). Blots were analyzed using 1D gel image analysis (TotalLab, Newcastele, UK). GAPDH was used as a housekeeping gene.

### Immunohistochemistry

Core samples were fixed overnight in 4% paraformaldehyde and embedded in paraffin after tissue processing. Five μm sections were made using the Leica HM360 microtome (Leica, Diegem, Belgium). Before staining, tissue was deparaffinated and rehydrated. Afterwards, antigen retrieval was performed by boiling the slides for 20 min in TRIS-EDTA buffer (pH = 9) and cooled down to room temperature. Slides were washed 3 times for 5 min in PBS and the last time with PBS-0.05% Tween after which the endogenous peroxidases were blocked for 20 min in 0.15% hydrogen peroxide in PBS-0.05% Triton. Slides were washed and blocked with 2% bovine serum albumin (BSA) in PBS-0.05% Triton for 30 min. Afterwards, slides were incubated overnight with the primary antibody in 1% BSA/PBS (VDR: 1/2000, SantaCruz Biotechnology; CYP27B1 1/1000, Abcam; CYP24A1 1/500, SantaCruz Biotechnology, cathelicidin: 1/100, SantaCruz Biotechnology). Next, slides were washed as previously described and incubated with secondary antibody for 40 min at room temperature (SuperBoost poly HRP, Thermofisher). After washing, cells were incubated with activated DAB solution (0.05% DAB/0.015% H_2_O_2_/0.01 M PBS pH 7.2) and washed. Slides were then counterstained with Mayer’s Hematoxylin for 10s. and rinsed for 10 min under running tap water and collected in distilled water after which slides were dehydrated and mounted with DPX mounting medium (VWR, Oud-Heverlee, Belgium). Slides were scanned and pictures were taken for visualization.

### Immunofluorescence

Tissue fixation was similar as for immunohistochemistry. Antigen retrieval was done in citrate buffer + 0.1% triton for 20 min after which the samples were allowed to cool down for another 20 min. Slides were washed in distilled water for 3 min and blocked with Bloxall (Lab concult, Schaarbeek, Belgium) for 15 min and the endogenous peroxidases were blocked for 30 min in 0.3% H_2_O2/5% goat serum/ TBS-Triton 0.1%. Slides were then washed 3 times with TBS-Tween 0.1% (TBST) and incubated overnight at room temperature with the primary antibody against p63 (Agilent, Santa Clara, California, USA). The morning after, slides were washed 3 times in TBST and the secondary antibody (SuperBoost™ Goat anti-Mouse Poly HRP, ThermoFisher) was added to the slides for 40 min. Slides were washed again 3 times with TBST and the tyramide signal amplification (TSA) dye 647 diluted in borate buffer was added to the slides for 10 min. Slides were washed twice in TBST and once in distilled water before antibodies were removed from the slides by boiling the slides for 20 min in TRIS-EDTA buffer. Slides were allowed to cool down and washed once in TBST. Slides were blocked in 5% goat serum/TBST. Next, the second primary antibody (VDR, Santa Cruz biotechnologies) was added to the slides for 1 h at room temperature. The secondary antibody was added as described above and after washing, the TSA dye 488 was added to the slides diluted in borate buffer for 10 min. Slides were washed once and cells were counterstained with DAPI and mounted using fluorescent mounting medium (Dako, Agilent). Background subtraction was applied to improve visualization.

### Statistics

An unpaired T-test or Mann Whitney-U test was used depending on the normality of the data. Normality was checked using the Shapiro-Wilk test.

Statistical analysis was performed in Graphpad Prism 8.0.

## Results

### MicroCT data - surface density

**Fig. 1:**
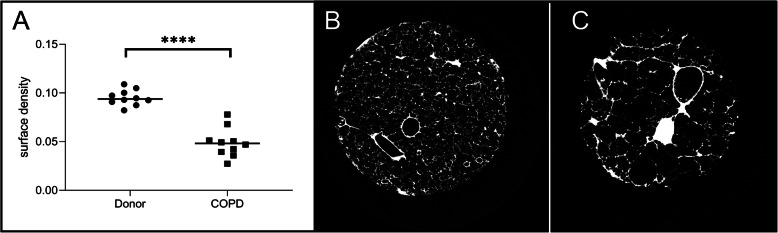
COPD cores show clear emphysema **a**. Surface density is significantly lower in cores from COPD explant lungs compared to cores from unused donor lungs. Representative images of a μCT from a core from an unused donor lung (**b**) and a COPD explant lung (**c**). Emphysema is clearly present in the core from the COPD explant lung. *N* = 10 per group **** *p* < 0.0001 Horizontal line represents the median value

All patients were clinically diagnosed with COPD. As emphysema is often unequally distributed over the lung, we measured surface density (3D measure for Lm, a measure for emphysema) based on μCT to ensure that cores used for expression analysis were truly representative samples. As expected, surface density was significantly lower in COPD lung cores compared to cores from unused donor lungs (*p* < 0.0001). Representative images of a μCT from both an unused donor lung and of a COPD lung core are shown in Fig. 1 in which the parenchyma of the unused donor lung looks normal while the parenchyma of the core from a COPD explant lung show extensive damage corresponding to emphysema. Total airway volume was significantly lower in COPD lung cores compared to cores from unused donor lungs (supplementary figure 1).

### CYP27B1 expression and localization in lung tissue

**Fig. 2:**
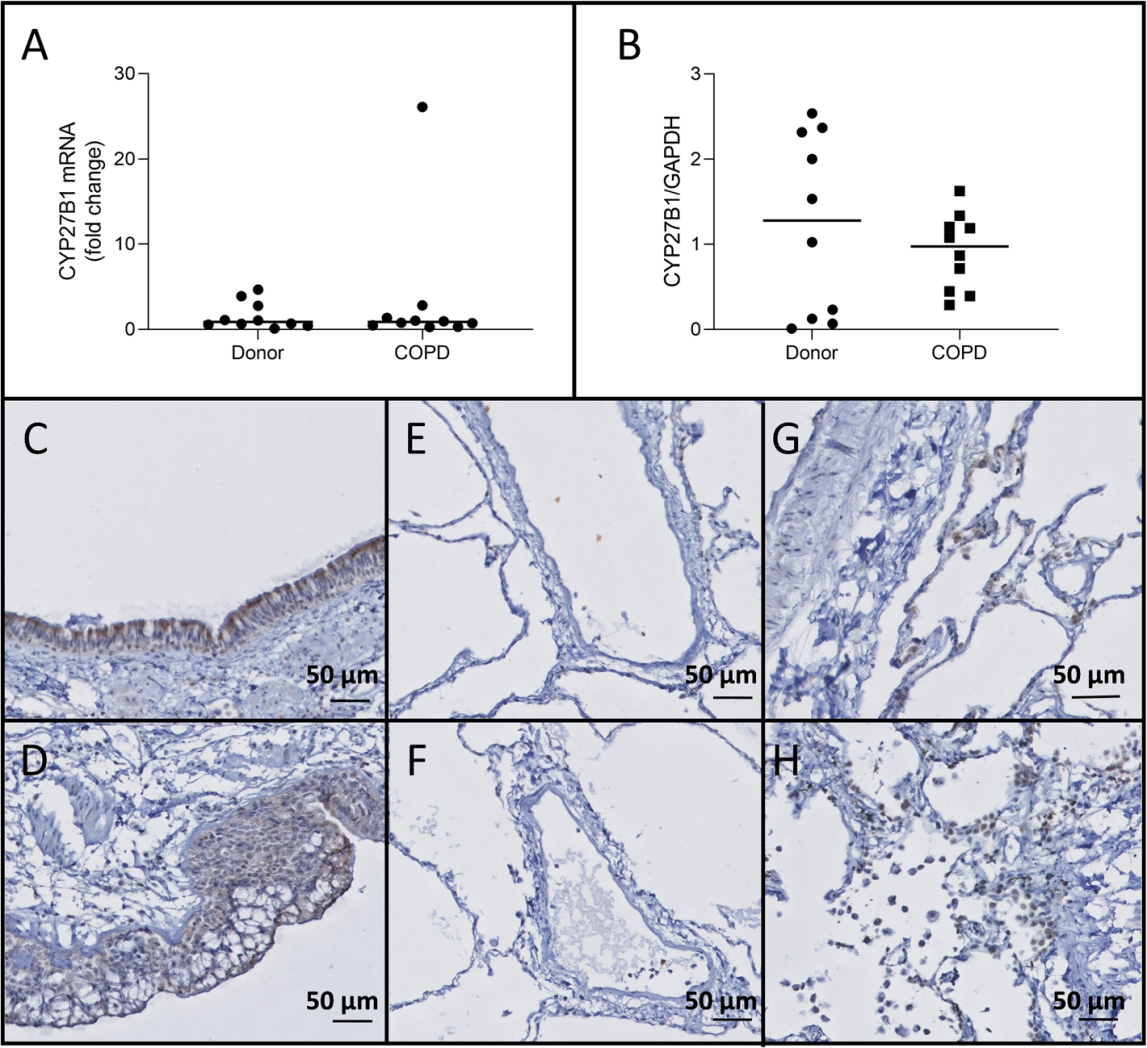
CYP27B1 expression No significant differences in CYP27B1 expression were detected between COPD explant tissue or tissue from unused donor lungs nor on mRNA level (**a**), nor on the protein level (**b**). CYP27B1 stained positive in lung epithelial cells of both unused donor lungs (**c**) and COPD explant lungs (**d**), while no staining was observed in endothelial cells of both unused donor lungs (**e**) and COPD explant lungs (**f**) and immune cells staining was inconclusive for both unused donor lungs (**g**) and COPD explant lungs (**h**). *N* = 10 Representative images are shown Horizontal line represents the median value

There were no significant differences in CYP27B1 expression between unused donor lungs and COPD explant lungs on mRNA level (*p* = 0.97)(Fig. 2a) and protein level (*p* = 0.40)(Fig. 2b). CYP27B1 stained positive in all layers of the bronchial epithelium and submucosal glands of both unused donor lungs and COPD explant lungs (Fig. 2c-d). CYP27B1 expression was absent in the endothelium (Fig. 2e-f), whereas staining of the immune cells remained inconclusive (Fig. 2g-h).

### CYP24A1 expression and localization in lung tissue

**Fig. 3:**
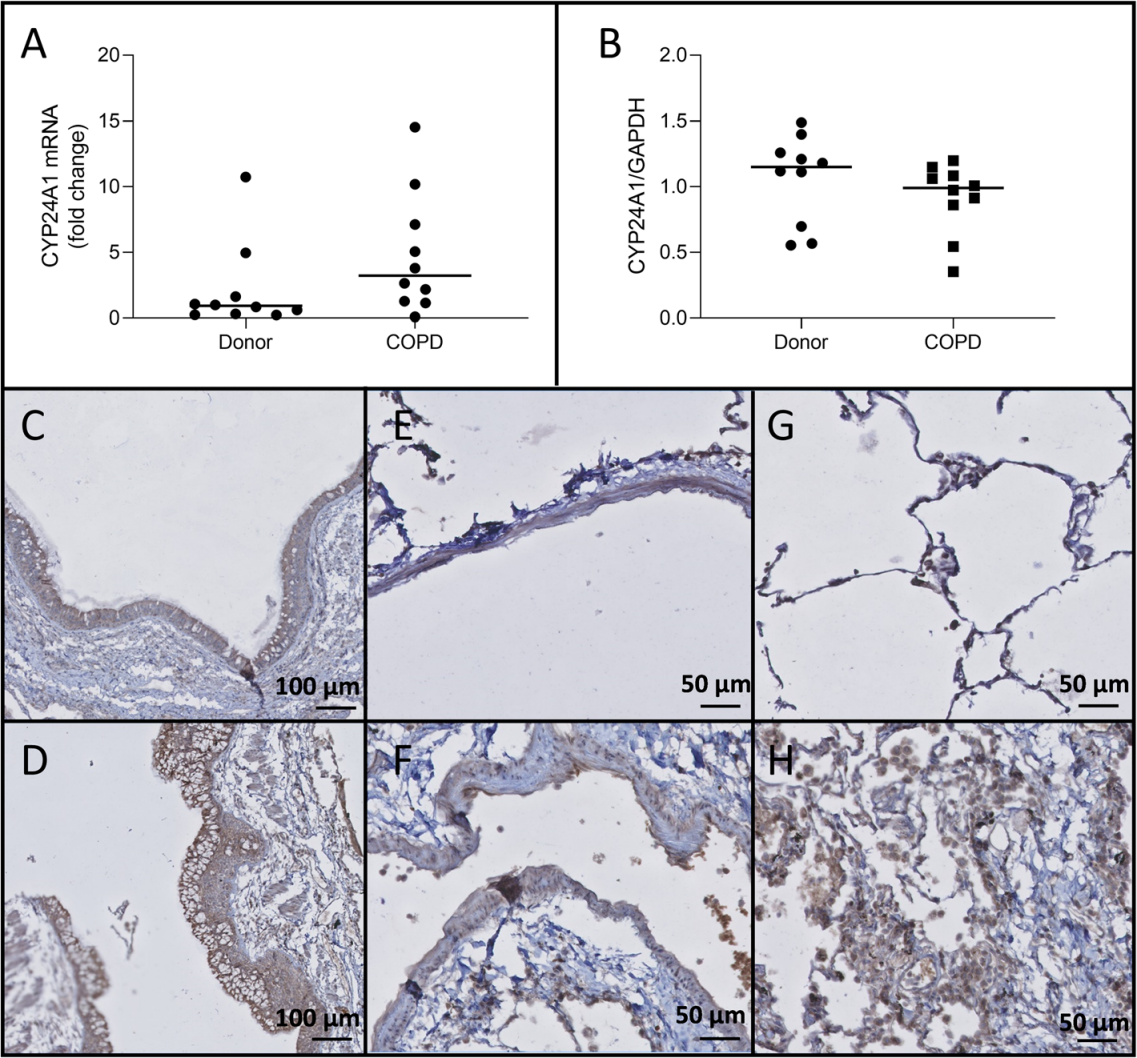
CYP24A1 expression **a**. No significant difference in CYP24A1 mRNA levels was observed between COPD explant tissue and tissue from unused donor lungs (*p* = 0.07). **b**. CYP24A1 protein expression was not altered in COPD explant tissue compared to tissue from unused donor lungs. CYP24A1 staining in bronchial epithelium of an unused donor lung (**c**) and COPD explant lung (**d**). Positive endothelial staining in an unused donor lung (**e**) and COPD explant lung (**f**). Positive immune cell staining in an unused donor lung (**g**) and COPD explant lung (**h**). Representative images are shown. *N* = 10 Horizontal line represents the median value

There was a non-significant trend towards an increase in CYP24A1 mRNA expression (*p* = 0.06) in COPD explant lungs, but this trend was not present on the protein levels (*p* = 0.30)(Fig. 3a-b). On immunohistochemistry, epithelial cells, submucosal glands and immune cells stained positively for CYP24A1 (Fig. 3c-d and g-h). In contrast to CYP27B1, the endothelial cells stained highly positively for the deactivating enzyme (Fig. 3e-f).

### Vitamin D receptor expression and localization in the lung

**Fig. 4:**
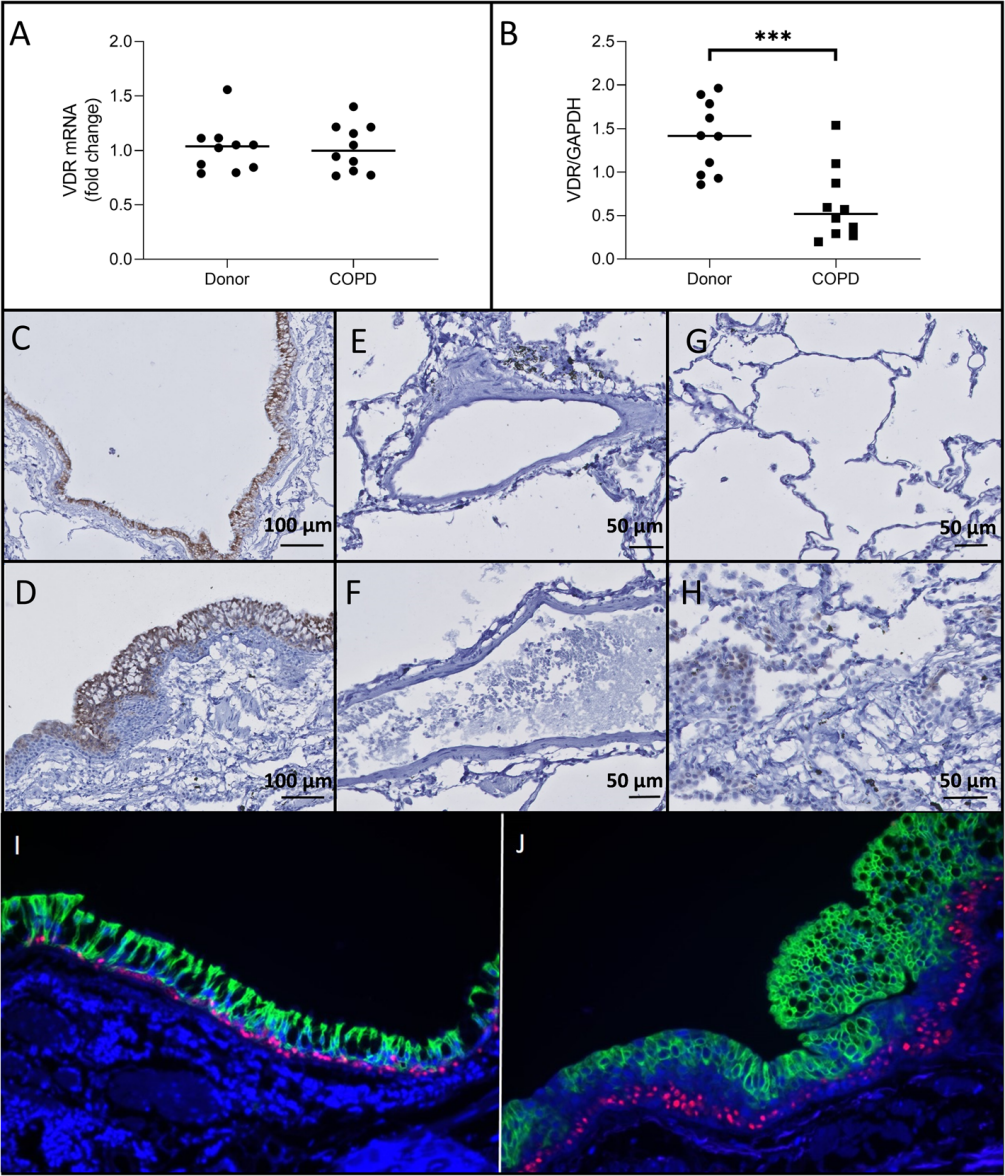
VDR expression **a**. No significant difference in VDR mRNA levels was observed between COPD explant tissue and tissue from unused donor lungs. **b**. VDR protein expression was significantly lower in COPD explant tissue compared to tissue from unused donor lungs (*p* = 0.0007). VDR is expressed in lung epithelial cells from unused donor lungs (**c**) and COPD explant lungs (**d**) and a light staining is observed in some immune cells of COPD patients (**h**) and unused donor lungs (**g**), but not in lung endothelial cells neither from unused donor lungs (**e**), nor from COPD explant lungs (**f**). Moreover, VDR expression in lung epithelial cells is restricted to the apical cells (green), while lung basal cells (p63, red) are negative for VDR) (**i**: unused donor lungs, **j**: COPD explant lung). Representative images for the stainings are shown. *N* = 10

There was no significant difference in VDR mRNA expression between unused donor lungs and COPD explant lungs (*p* = 0.97)(Fig. 4a). On the protein level, VDR expression was significantly lower in COPD explant lungs as shown on Western Blot (*p* = 0.0007)(Fig. 4b)(Supplementary Figure 2) although staining intensities on immunohistochemistry did not indicate different expression (Fig. 4c-d). Strong staining of VDR was limited to the bronchial epithelium and submucosal glands (Fig. 4c-h). However, and in contrast to CYP27B1 and CYP24A1, VDR staining in the bronchial epithelium was restricted to the apical cells, particular in the COPD patients. Co-staining of VDR with p63, a marker for basal cells, revealed that basal cells were negative for VDR (Fig. 4i-j). The limited number of immune cells stained occasionally positively but weakly.

### Cathelicidin expression in lung

**Fig. 5:**
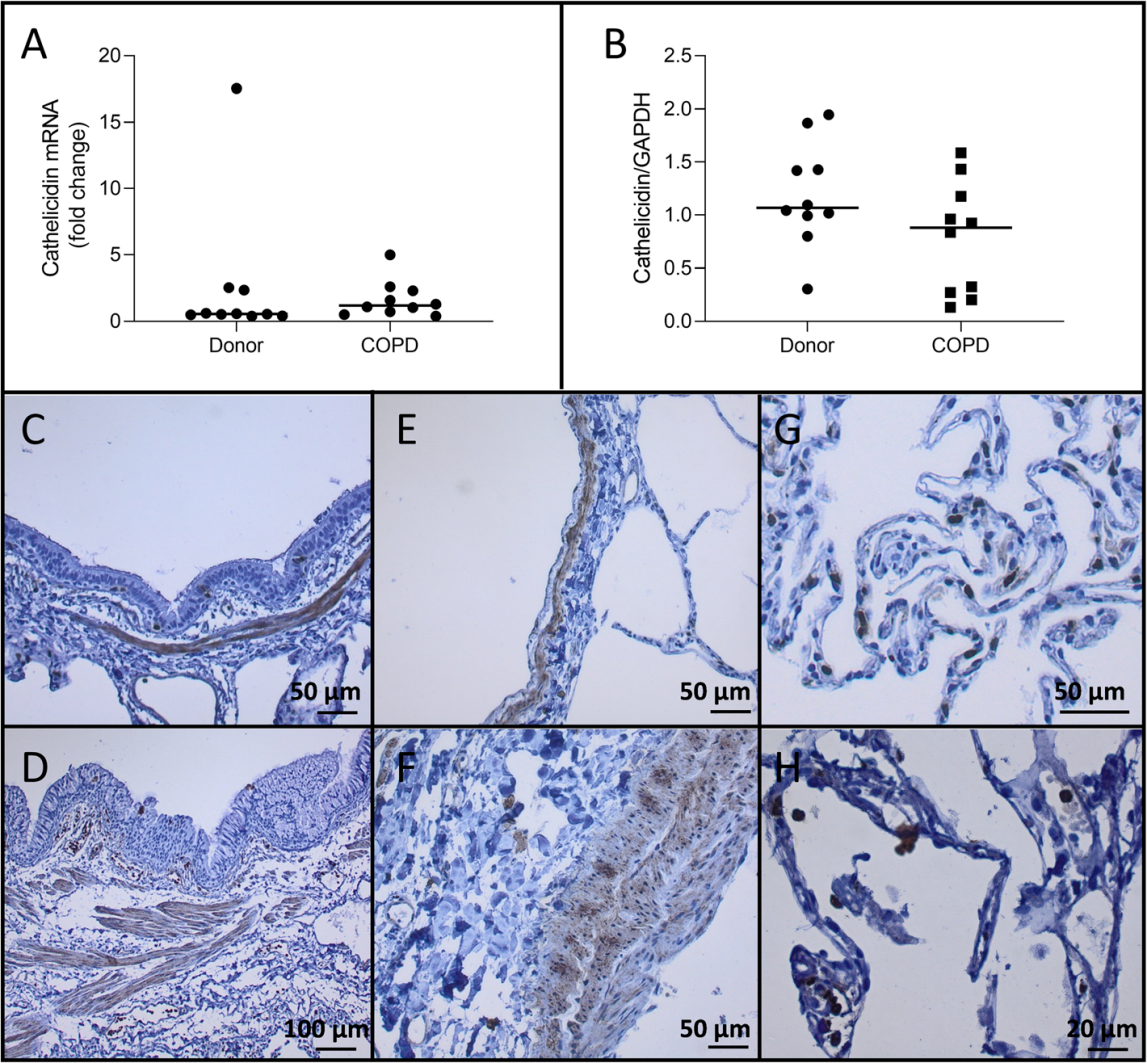
Cathelicidin expression No significant difference in cathelicidin mRNA levels were observed between COPD explant tissue and tissue from unused donor lungs (p = 0.35). **b**. Cathelicidin protein expression was not different in COPD explant tissue compared to tissue from unused donor lungs (p = 0.09). Negative cathelicidin staining in bronchial epithelium with positive smooth muscle layer of an unused donor lung (**c**) and COPD explant lung (**d**). Negative endothelial staining with positive smooth muscle layer in an unused donor lung (**e**) and COPD explant lung (**f**). Positive immune cell staining in an unused donor lung (**g**) and COPD explant lung (**h**). Representative images are shown. *N* = 10 Horizontal line represents the median value

No significant difference in mRNA expression of cathelicidin (*p* = 0.35) was detected between tissue from unused donor lungs and tissue from COPD explant lungs (Fig. 5a) while a non-significant reduction in the protein level of cathelicidin (*p* = 0.09) was observed between COPD and healthy donor lungs (Fig. 5a - b). Smooth muscle cells surrounding airways and blood vessels and immune cells stained positive for cathelicidin while endothelial cells and bronchial epithelial cells stained negative (Fig. 5c - h).

## Discussion

This is the first study comprehensively examining expression levels (mRNA and protein) and localization of VDR, CYP27B1 and CYP24A1 in explanted lung tissue of COPD patients and of unused donors. While no differences in expression were observed between unused donor lungs and COPD lungs for both CYP27B1 and CYP24A1, VDR protein levels were significantly decreased in COPD lungs compared to unused donor lungs. The reduction in VDR protein expression of COPD lungs did not translate into an altered expression of cathelicidin, a direct VDR dependent target gene. Interestingly, VDR localization was restricted to the apical lung epithelial cells while it was completely absent in the basal cells or stem cells of the epithelium. CYP27B1 and CYP24A1 were localized in all lung epithelial cells including the basal fraction, while in endothelial cells, only CYP24A1 was present.

Even though multiple studies have investigated the effect of oral vitamin D supplementation in COPD, studies evaluating expression of key vitamin D-related genes and proteins in lung tissue of COPD patients are lacking. Recent data clearly demonstrated that COPD and asthma patients have different 1.25(OH)_2_D inducible gene expression signatures in peripheral tissue compared to controls [18]. In our study, CYP27B1, which is an essential enzyme to convert 25(OH)D to the active form 1,25(OH)_2_D, was stably expressed in bronchial epithelial cells but inconsistently present in immune cells. It indicates that vitamin D can be locally activated within airway epithelial cells but also by residing immune cells, which is in agreement with earlier reports [19, 20]. We did not identify different expression profiles between COPD and controls. In literature, contradictory findings concerning the expression levels of CYP27B1 in lung cells have been reported and these discrepancies are likely related to a wide variation in methodology and cellular material. Liu et al. were the first to describe that toll like receptor triggering of macrophages and monocytes with *Mycobacterium tuberculosis* increased CYP27B1 mRNA expression [20]. In the 16HBE cell line, we recently described an upregulation of CYP27B1 mRNA in bronchial epithelial cells after cigarette smoke extract exposure [21]. By contrast, using differentiated primary human airway epithelia cultures at the air-liquid interface from healthy individuals, Buonfiglio et al. reported a decrease in CYP27B1 mRNA expression upon treatment with cigarette smoke extract as did Mulligan et al. when using primary sinonasal epithelial cells from healthy subjects and patients with chronic rhinosinusitis [22, 23]. CYP27B1 mRNA expression in airway epithelial cells was unaltered after exposure to TNFα/IL1β [24]. Overall, these data indicate that the potential of local 25(OH)D activation is relatively preserved in lungs of patients with severe COPD.

Vitamin D inactivation by CYP24A1 is also preserved in the lungs of COPD patients. In contrast to CYP27B1 and VDR expression, expression of this enzyme was not limited to bronchial epithelial cells, but also endothelial cells and immune cells stained positive. Although a trend towards higher CYP24A1 mRNA expression in COPD samples was detected in our study, this was not confirmed on protein level. Different studies have found an upregulation of CYP24A1 mRNA in bronchial epithelial after infection or triggered by inflammatory cytokines, others did not observe important changes [13, 21, 24, 25]. One study even reported a decrease in CYP24A1 mRNA level in tissue from COPD patients compared to control tissue. As these subjects had significantly lower serum 25(OH)D levels [26] compared to the serum 25(OH)D levels in our group, it might explain why CYP24A1 expression levels were comparable between both our groups.

VDR is essential as a receptor and co-factor for the expression of target genes. Although no difference in VDR mRNA expression was detected in our study, protein levels of VDR were significantly lower in COPD explant lung tissue compared tissue of unused donor lungs. Reduced VDR protein expression in COPD lung tissue may have important consequences, but VDR immunohistochemistry stained equally positively for COPD and control tissue in the apical layer of the epithelium. A potential explanation for the discrepancy between VDR protein expression and immunohistochemistry staining may come from the unique localization of VDR at the apical layer of the bronchial epithelium in combination with the lower total airway volume in COPD samples as compared to healthy controls (supplementary figure 1). As the downstream signaling with expression of cathelicidin, was not reduced in COPD lung tissue, it indicates that VDR functioning was relatively well preserved, even in COPD lungs. As VDR expression is not only affected by inflammatory signals, including cigarette smoking, but also by vitamin serum levels, additional studies are needed to explore VDR expression in the context of COPD [27–29]. These studies should also focus on nuclear versus cytoplasmatic localization of VDR as Uh et al. reported that cigarette smoke inhibits VDR translocation in alveolar cell line [30]. Moreover, studies performing double staining of different epithelial cells (ciliated cells, goblet cells and club cells) with VDR might provide additional information on the localization of VDR in the apical epithelial cells. Interestingly, in the present study, VDR expression was found to be restricted to the apical epithelial cells while being completely absent in basal cells or stem cells of the epithelium. This may have important consequences for the treatment with vitamin D supplementation, and suggests that direct inhalation may offer an alternative and more powerful therapeutic option*.* In fact, several studies have shown that VDR expression and vitamin D are critical for fetal and neonatal lung formation [31, 32], but its potential role in the regeneration, differentiation or maintenance of a healthy epithelium is unclear. Further studies are needed to address these issues.

As activating and deactivating enzymes, receptors and co-factors may drastically impact on vitamin D mediated effects locally in the tissue, we selected cathelicidin as a readout. The gene encoding for cathelicidin is a direct target of vitamin D as it possesses a unique vitamin D responsive element in its promotor [14]. The cathelicidin protein is an antimicrobial peptide with antibacterial, antiviral and anti-inflammatory potential [13, 15, 16]. We found that cathelicidin was equally expressed in lung tissue of COPD patients and controls. Although it is still possible that other structural cells and inflammatory cells in COPD have compensated for reduced expression levels of cathelicidin in the airway epithelium, it indicates that the vitamin D signaling pathway in the lung is relatively well conserved and still amendable for interventions.

Currently, vitamin D is given to COPD patients as an oral supplement. However, as most key enzymes are present in the airway epithelium, direct administration through inhalation may potentially improve its anti-inflammatory effects and overcome the dose limitation, inactivation and toxicity of systemic supplementation [33]. Proof of concept was found in a hamster model of acute lung injury in which local vitamin D application reduced neutrophil recruitment in the broncho-alveolar lavage [34]. Furthermore, it is tempting to speculate that by the unique expression of CYP24A1 in the vascular endothelium, systemic vitamin D supplementation may have failed in the general COPD population [35–37], as 25(OH)D and 1.25(OH)_2_D are potentially inactivated prior to their translocation to airways and tissue residing immune cells [38]. This issue definitely warrants future research.

One major limitation of our study is that we only focused on a random core of the parenchyma and addressed a limited number of key enzymes and target proteins, thereby neglecting potential disease heterogeneity and the full complexity of the vitamin D pathway. New studies taking the full transcriptome into account as well as different disease stages, are needed to confirm our findings. Another limitation of this study is that we do not have all donor characteristics. In particular, the lack of detailed smoking history, 25(OH)D levels and duration of supplementation status may have influenced our conclusions. Moreover, the relative small sample size may explain the lack of differences in expression. Recently, a large study analyzing gene expression data of key enzymes including CYP24A1, CYP27B1 and VDR in lung tissue partially confirmed our findings on mRNA level [18], but protein and localization data of a larger sample size are warranted.

## Conclusion

While no changes in mRNA and protein expression levels were detected for CYP27B1 and CYP24A1, protein levels of VDR were significantly lower in lung tissue of COPD patients compared to lung tissue of unused donor lungs. VDR, CYP27B1 and CYP24A1 were all expressed in bronchial epithelial cells, but VDR expression was limited to apical epithelial cells, while CYP24A1 was the only one to be expressed in endothelial cells. In conclusion, the unique expression profiles of key enzymes of the vitamin D pathway in lung tissue of end stage COPD patients confirms the potential of vitamin D in mediated anti-inflammatory and anti-microbial interventions in COPD, preferentially through inhalation.

## Supplementary information

**Additional file 1: Figure S1.** Core airway volume. Airway volume was significantly lower in cores from COPD tissue compared to cores from unused donor lungs (*p* = 0.023). Mann-Whitney U-Test *N* = 10.

**Additional file 2: Figure S2.** Protein expression. Representative images of CYP27B1 (A), CYP24A1 (B), VDR (C) and cathelicidin (D) protein expression. C = COPD, D = Donor.

## Data Availability

All data generated or analyzed during this study are included in this published article and its supplementary information file.

## Abbreviations

1,25(OH)_2_D: 1,25 dihydroxyvitamin D
25(OH)D: 25-hydroxyvitamin D
BSA: bovine serum albumin
COPD: Chronic Obstructive Pulmonary Disease
CYP24A1: 24-hydroxylase
CYP27B1: 1α-hydroxylase
DAB: 3,3′-Diaminobenzidine
DLco%: % predicted diffusion capacity
FEV1%/FVC: Tiffeneau index
FVC%: % predicted forced vital capacity
GAPDH: Glyceraldehyde-3-phosphate dehydrogenase
MOPS: 3-(N-morpholino)propanesulfonic acid
NFDM: Non-fat dry milk
PBS: Phosphate buffered saline
TBS: Tris buffered saline
TBST: Tris buffered saline-Tween
TLC%: % predicted total lung capacity
TSA: tyramide signal amplification
VDR: Vitamin D receptor
