# Supplementary figures and images for: Local expression profiles of vitamin D-related genes in airways of COPD patients
Source: Respir Res. 2020 Jun 3;21:137. doi: 10.1186/s12931-020-01405-0 (PMC7268690; doi:10.1186/s12931-020-01405-0)

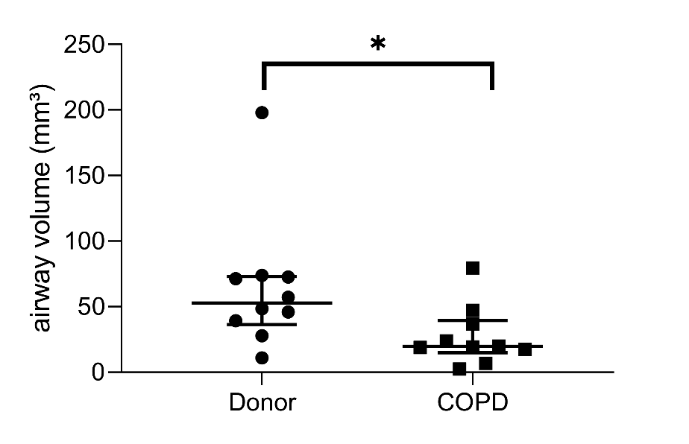

Supplement: Supplementary file 1 — Additional file 1: Figure S1. Core airway volume. Airway volume was significantly lower in cores from COPD tissue compared to cores from unused donor lungs (p = 0.023). Mann-Whitney U-Test N = 10. [file 12931_2020_1405_MOESM1_ESM.docx]

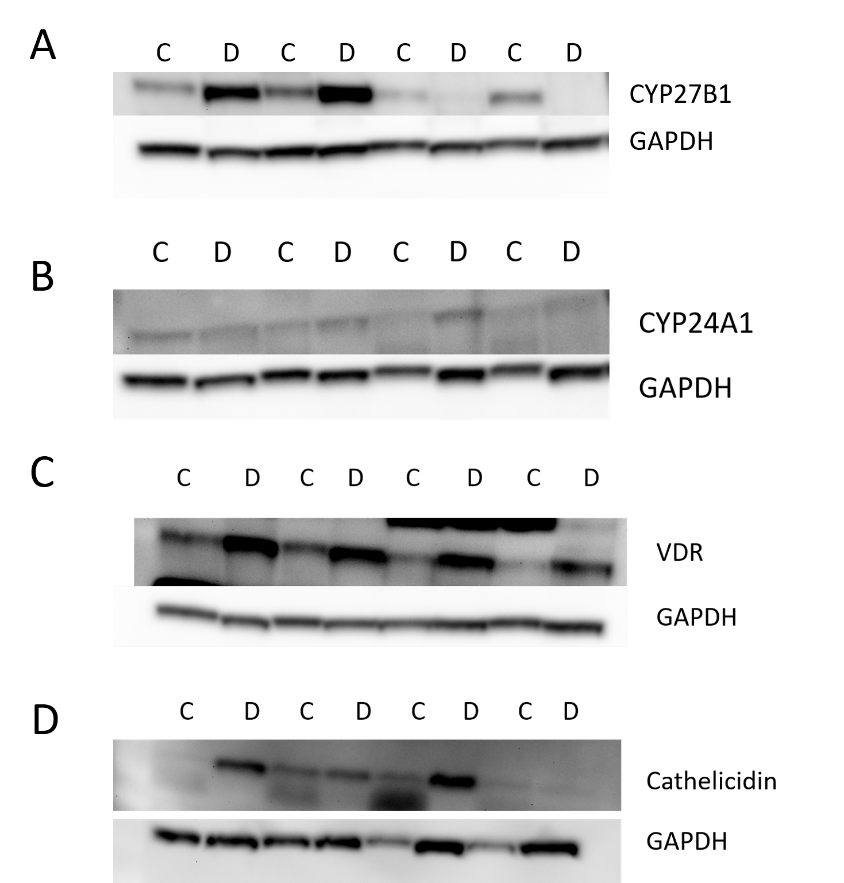

Supplement: Supplementary file 2 — Additional file 2: Figure S2. Protein expression. Representative images of CYP27B1 (A), CYP24A1 (B), VDR (C) and cathelicidin (D) protein expression. C = COPD, D = Donor. [file 12931_2020_1405_MOESM2_ESM.docx]
